# Does a 4–6 Week Shoeing Interval Promote Optimal Foot Balance in the Working Equine?

**DOI:** 10.3390/ani7040029

**Published:** 2017-03-29

**Authors:** Kirsty Leśniak, Jane Williams, Kerry Kuznik, Peter Douglas

**Affiliations:** 1 Centre for Performance in Equestrian Sport, Hartpury College, Gloucester GL19 3BE, UK; jane.williams@hartpury.ac.uk (J.W.); kerrykuznik@hotmail.co.uk (K.K.); 2 PE Douglas DWP, Ivybridge, Devon PL21 0NP, UK; peter.douglas@hotmail.com

**Keywords:** equine, hoof, shoeing, hoof angle, conformation, morphometric measurements

## Abstract

**Simple Summary:**

Hoof shape is linked to an increased risk of lameness in the horse and has been shown to adapt to different loading patterns associated with the workload and shoeing interval length. This study investigated how different measurements of the hoof wall and the hoof pastern axis angle changed with work in riding school horses, across a four to six week shoeing/trimming interval. The dorsal hoof wall, and weight bearing and coronary band lengths reduced in size post-shoeing/trimming. This, combined with the increase to the inner and outside hoof wall heights on the digital images despite trimming, suggests that shoeing/trimming increased the vertical orientation of the hoof during the shoeing interval investigated. At the same time, increases in the dorsal hoof wall angle, heel angle, and heel height occurred, promoting a more correct dorsopalmar balance. The changes observed are consistent with the workload of the horses studied. The results suggest that a regular farriery interval of no more than six weeks could prevent excess loading of the structures within the hoof, reducing long term injury risks through cumulative, excessive loading in riding school horses.

**Abstract:**

Variation in equine hoof conformation between farriery interventions lacks research, despite associations with distal limb injuries. This study aimed to determine linear and angular hoof variations pre- and post-farriery within a four to six week shoeing/trimming interval. Seventeen hoof and distal limb measurements were drawn from lateral and anterior digital photographs from 26 horses pre- and post-farriery. Most lateral view variables changed significantly. Reductions of the dorsal wall, and weight bearing and coronary band lengths resulted in an increased vertical orientation of the hoof. The increased dorsal hoof wall angle, heel angle, and heel height illustrated this further, improving dorsopalmar alignment. Mediolateral measurements of coronary band and weight bearing lengths reduced, whilst medial and lateral wall lengths from the 2D images increased, indicating an increased vertical hoof alignment. Additionally, dorsopalmar balance improved. However, the results demonstrated that a four to six week interval is sufficient for a palmer shift in the centre of pressure, increasing the loading on acutely inclined heels, altering DIP angulation, and increasing the load on susceptible structures (e.g., DDFT). Mediolateral variable asymmetries suit the lateral hoof landing and unrollment pattern of the foot during landing. The results support regular (four to six week) farriery intervals for the optimal prevention of excess loading of palmar limb structures, reducing long-term injury risks through cumulative, excessive loading.

## 1. Introduction

Equine distal limb lameness is commonly associated with poor foot conformation and hoof imbalance [1,2,3,4], with hoof-related lameness being a key cause of poor performance and early retirement in the sport [5,6] and as a pleasure horse [6,7,8].

Research has identified that the biomechanical function of the distal limb can alter as a result of changes in the hoof shape. Consequently, this influences the forces acting on the hoof’s structural components [9,10], as well as the distal interphalangeal joint (DIPJ) and proximal interphalangeal joint (PIPJ) moments [6], the leverage on the toe at breakover, and the forces acting on the navicular bone [2]. An example of such biomechanical influences are those which facilitate the breakover of the stride, achieved through the shortening of the toe. The alternation in hoof orientation achieved through the shortening of the toe is suggested to result in improved angulation between the proximal and middle phalanx, thus elevating the position of the navicular bone and consequentially reducing the loading of the deep digital flexor tendon (DDFT) [11,12]. The principles behind this biomechanical influence have been utilised by veterinarians and farriers in the application of heel wedges or rocker shoes for the treatment of conditions such as DDFT tendinopathies and navicular syndrome [13]. Hoof conformation refers more to the geometric morphology of the static foot [14,15]. The term balance is recognised as not only a consideration of the geometric shape of the hoof, but also the way in which this interacts with the rest of the limb and the ground with which it is in contact [15]; this includes dorsopalmar balance, which refers to hoof pastern axis alignment. Consequently, the impact of trimming and shoeing the feet can affect the health, performance, and longevity of the equine athlete [8]. Therefore, the principal role of the farrier is to balance the feet of the horse to facilitate optimal movement, prevent injury, and to improve performance [8,16]. Farriery should always include trimming the feet and can include applying horse shoes. The majority of modern horses are shod to cope with the demands of their workload, to enhance performance, and to extend career longevity [8]. Despite advances in research, farriery largely remains a profession based on traditional empirical craftsmanship, rather than scientific evidence [4,17]. Regular farrier treatments are recommended to maintain or improve the athletic performance capacity of the horse [6,18]. However an ‘optimal’ shoeing/trimming interval has not been defined. Moleman et al. [6] suggested that the shoeing/trimming interval length should be determined for individual horses by their farrier, to meet their specific needs. The interval length may also be influenced by the knowledge, understanding, and potentially financial constraints of horse owners and keepers.

Anecdotally, within the equine industry, shoeing/trimming interval lengths are commonly between four to eight weeks. However, intervals can vary beyond eight weeks, especially within the leisure horse population. Within research, the length of recommended shoeing/trimming intervals vary between four to six weeks, or six to eight weeks [4,6,7,18,19]. The majority of studies to date have evaluated changes in the hoof associated with eight week intervals [4,8]. An eight week interval has been connected with increases in the dorsal hoof wall length (DHWL) and a reduction in the dorsal hoof wall angle (DWHA), which places the DIPJ under increased strain [6]. If the aim of farriery is to restore the balance of the hoof, correct conformational defects, and optimise distal limb biomechanics, then the ideal shoeing/trimming interval should facilitate consistency in the loading of the foot, and by default, the associated structures of the distal limb. Therefore, shorter intervals between four to six weeks may benefit the horse if they can be shown to limit changes within the foot, and by association, the distal limb. Currently, there is a paucity of evidence-based knowledge concerning the linear and angular changes that occur within the hoof associated with shoeing/trimming intervals, in order to confirm the potential benefits of this practice (four to six week interval) [6,20]. Therefore, the aim of this study was to determine how linear and angular hoof morphometric measurements varied pre- and post-farriery with a four to six week shoeing/trimming interval, in horses which were free from lameness and regularly shod/trimmed. We hypothesised that the foot of the horse would be more symmetric and balanced, and that the hoof pastern axis (HPA) would present in correct alignment post-farrier treatment compared to pre-farrier treatment, but that the differences observed would not be significant pre- and post-farriery.

## 2. Method

Twenty-six horses of mixed breed, gender, age (12 ± 9.9 years), and height (157 cm ± 2.3 cm), which did not display any stereotypic behaviours and were resident at Hartpury College, Gloucestershire, UK, were selected for inclusion in the study. All subjects were on loan for use within the College’s riding school and were subjected to the same exercise and management regime: two 45-minute flat, jump, or lunge lessons per day (ProWax, Andrew Bowen, Singleton, Lancashire, UK), with one day off per week, and stabled (rubber matting and shavings) with restricted grass turnout. The horses included in the study were deemed in good health and functionally fit for work by experienced equestrian professionals who had daily access to an equine veterinary surgeon, to examine horses presenting with lameness. The horses declared unfit for work by the veterinary surgeon were removed from the study. Each individual received regular farrier treatment (hot shod; full set or front shoes) by one main farriery team (WCF (Worshipful Company of Farriers) qualified), at shoeing intervals between four and six weeks. Farriery was performed by one of four farriers under the direction and supervision of a lead farrier, to promote a consistent approach. All horses had been previously exposed to farrier treatment and were not undergoing any corrective farriery. Ethical approval for the study was granted by the University of the West of England (Hartpury) Ethics Committee (Project Identification Code: ETHICS2016-03).

Data were collected between September and December, when horses presented for their next farrier treatment. Digital images were taken of each horse’s left forelimb and hoof, to enable a comparison of morphometric measurements of key anatomical features and angles (Table 1) pre- and post-farriery treatment, with old and new or refitted shoes, respectively [6,21,22].

### 2.1. Protocol

**Figure 1:**
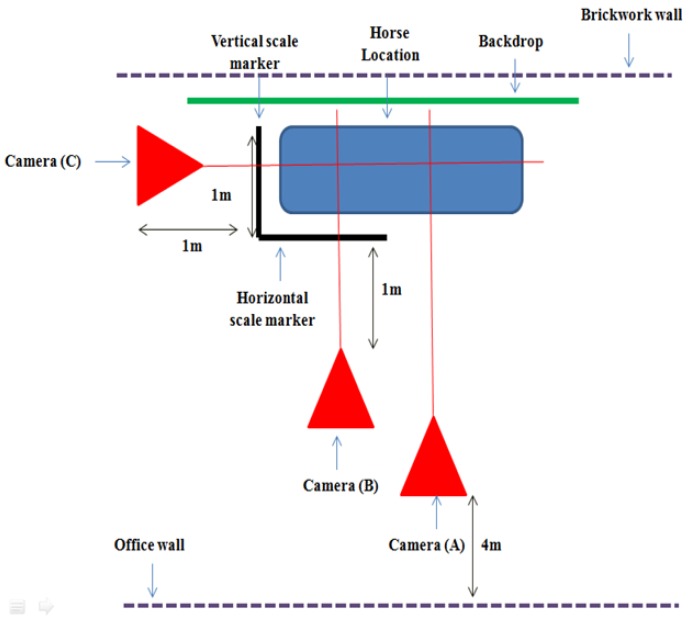
Layout of cameras, scale markers, backdrop, and positioning of the horse for digital image collection.

Prior to data collection, horses had their rugs removed and limbs cleaned. Key anatomical landmarks were used to orientate the placement of circular markers on the left forelimb and hoof, to ensure that the same anatomical regions were measured within all of the horses (Table 1) and to facilitate subsequent measurements from the digital images. Horses were stood square, with equal weight bearing on all four limbs, on a concrete surface and within a calibrated and marked out grid, to enable repetition and accuracy of positioning. Scale markers were used to mark out the area, to allow for image calibration and accurate digital measurement [2]. Horses also stood in front of a black board in the marked out area, to provide a contrast with the foot and forelimb [21]. Lateral and anterior digital photographic images of the left forelimb were obtained by using three digital cameras (Panasonic DMC-FZ45; 14.1 MP, Panasonic, Bracknell, UK and Ireland), as shown in Figure 1. For the acquisition of the lateral view of the limb, camera A was attached to a tripod (Velbon DV-7000, Velbon, Maidenhead, UK) (height 170 cm, base wide 82 cm), which was positioned centrally to the marked out area and four metres (m) back from the wall. Lateral images of the hoof were obtained by camera (B) at a 0.3 zoom, positioned just above ground level and one metre back from the horizontal scale marker, midway in line with the marked out grid on the floor laterally in line with the hoof. Camera (C) was positioned one metre back from the vertical scale marker at a 0.3 zoom and held just above ground level, in order to take images of the dorsal hoof aspect from an anterior view of the horse [6]. Images from camera A confirmed that horses were standing square; the images from cameras B and C were used to facilitate measurements.

### 2.2. Data Processing and Analysis

The photographic images were imported into Dartfish™ software version 7 (Dartfish, Fribourg, Switzerland), to facilitate the angular and linear measurements outlined in Table 2, Table 3 and Table 4. Hoof angle displacement, the fetlock joint angle, and vertical displacement were determined by plumb line measurements. All measurements were repeated three times for each photographic image and their mean was calculated. Mean data were exported to Microsoft Excel, Version 15 (Microsoft, Washington, DC, USA), for categorical organisation, and to allow the mean and standard deviation to be determined for each variable, across the cohort. Van Heel et al. [4] utilised force plate determination of Centre of Pressure (CoP) to propose an equation which successfully predicts the shift of CoP relative to the toe, from the hoof measurements.

Van Heel et al.’s [4] predictive equation for CoP is as follows:

∆d = a_old._cosα_old_ − a_new._cosα_new_ where, in the current study, a_new_ is the length of the dorsal hoof wall post-farriery, α_new_ is the hoof angle post-farriery, a_old_ is the length of the dorsal hoof wall pre-farriery (after four to six weeks), and α_old_ is the hoof angle pre-farriery. d is the predicted location of CoP at midstance, relative to the point of rotation at the toe.

Using the Van Heel et al. [4] equation, the shift in distance that occurred for the CoP after farrier treatment was calculated pre- and post-farriery treatment for each individual horse and to provide mean measurements for pre- and post-farriery CoP for the cohort. A series of paired t-tests analysed whether differences existed between the variables examined from the pre- to the post-farrier treatment periods using Statistics Package for the Social Sciences (SPSS) Version 20 (IBM Corp., Armonk, NY, USA). Paired t-tests were also used to identify whether differences were present between the medial and lateral hoof angles and lengths in the foot, both prior to shoeing, and after the horses had been shod. A series of Pearson’s product correlations examined whether any linear relationships occurred between the variables measured both pre- and post-farriery treatment. Significance was set at *p ≤* 0.05.

## 3. Results

### 3.1. Hoof Measurements 

Significant differences were reported after shoeing for the majority of the lateral view, anterior view, and HPA measurements (Table 5).

### 3.2. Medio-Lateral Variation

**Figure 2:**
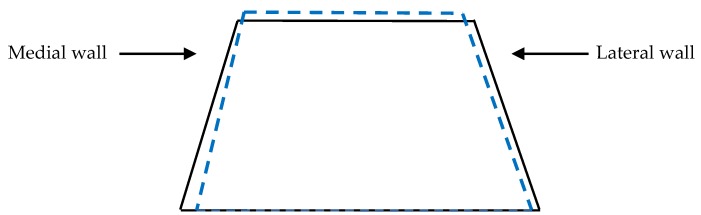
Pre-trimming (solid black line) and post trimming (blue dotted line); note the more upright angle of the medial wall, acutely angled lateral wall, and increased wall lengths.

Significant medio-lateral variation was reported between the linear and angular measurements in the hoof (Figure 2). The mean LDHWL (3.72 ± 0.68 cm) was found to be 4% longer (*p* = 0.01) than the mean MDHWL (3.57 ± 0.69 cm), prior to farriery. Medio-lateral variation in DHWL demonstrated an increased significant difference after shoeing (*p* = 0.0001), with the mean LDHWL (4.09 cm ± 0.80 cm) being 7% longer than the mean MDHWL (3.84 ± 0.74 cm). Similar results occurred for the hoof angle; significant differences were found between the mean MHA and mean LHA prior to farriery (*p* = 0.0001), with the MHA (78.96° ± 5.81°) being 7% greater than the LHA (73.17° ± 4.20°). Again, a more significant difference was recorded after shoeing (*p* = 0.0001), with the direction of difference remaining consistent, with MHA (80.80° ± 5.41°) being 10% greater than LHA (72.79° ± 4.07°).

### 3.3. Centre of Pressure

The calculated shift in the CoP distance found that the predicted CoP was located, on average, 0.5 cm back from the point of rotation of the toe across the cohort after shoeing.

### 3.4. Measurement Correlations

Significant correlations were found between twenty-eight pairs of variables measured prior to farriery treatment, and these ranged in strength and direction (Table 6). After shoeing, the number of correlations within the morphometric measurements of the hoof reduced, with only twenty-three pairs shown to be significantly associated (Table 6). 

## 4. Discussion

Correct (balanced) hoof conformation is essential to prevent lameness [24]. For example, Wright [25] recorded that 45.2% of horses demonstrating lameness were mediolaterally imbalanced. Balance is related to the shape and size of the hoof and is influenced by the relationship between the skeletal structures of the limb and the hoof [1,14]. Therefore, to achieve appropriate hoof balance, an understanding of the interaction between hoof conformation, movement, and the athletic activity of the horse is required [19].

DHWL was reduced by less than expected in the horses studied. Kummer et al. [8] reported that the DHWL shortened, on average, by 1 cm (*n* = 40) during an eight to ten week shoeing interval. Extrapolation from these results would suggest that the four to six week interval applied here would have been expected to reduce DHWL by 0.5 to 0.75 mm, which is much greater than was found (0.25 ± 0.97 cm). The discrepancies between these results are likely reflective of the differing study durations (Kummer et al. [8] collected repeated data over 12 months), the addition of a biotin supplement to Kummer et al.’s [8] study population, and the differing load and variation of work requirement (dressage and show jumpers in to Kummer et al.’s [8] study, compared to riding school horses here).

DHWA is defined as the angle formed at the junction of the DHW and the weight bearing surface of the foot [8,26] and a correct angle is essential to achieve an optimal hoof pastern axis. Despite still being documented in practitioner aimed literature until recently, the historic ideal DHWA of 45° for the front feet has been contradicted in science and practice [26,27]. Variability in DHWA is reported [28]; however, it is widely recognised that the ideal angle for the front feet should range between 50° to 55°. This is within the same range as that suggested for the hind feet [27,29], although wide variation (45° to 60°) in forelimb DHWA exists within the literature [2,8,10,22,30]. The DHWA mean values pre- and post-farriery fall within ‘normal’ DHWA ranges: 48.6° to 58.4°. The range reported here is comparable with previous research: Thomason et al. [31] (*n* = 10) reported a DHWA ranging from 48° to 57° and a mean angle of 51.8°; and Dyson et al. [22] (*n* = 19) reported a mean DHWA of 52.4°, ranging 43.4°–64.7°.

The decrease in DHWA associated with a four to six week interval (2.26° decrease, approximately 0.94° per two weeks) is analogous to the 3.3° decrease reported by Moleman et al. [6] across an eight week shoeing interval, but is greater than the 2.5° decrease (approximately 0.57° per two weeks) reported across an 8–10 week shoeing interval by Kummer et al. [8]. Force distribution in the hoof is related to DHWA, with more acute angles increasing loading in the heels. For example, a 39° DHWA angle results in 75% of loading weight within the heels, compared to 57% loading when the angle is increased to the ‘normal’ 55° [32]. Therefore, longer shoeing/trimmer intervals which result in decreased DHWAs will increase palmar loading, resulting in the weakening and collapse of the heels, and will amplify loading of the suspensory apparatus, leading to an increased susceptibility to injury [33,34].

**Figure 3:**
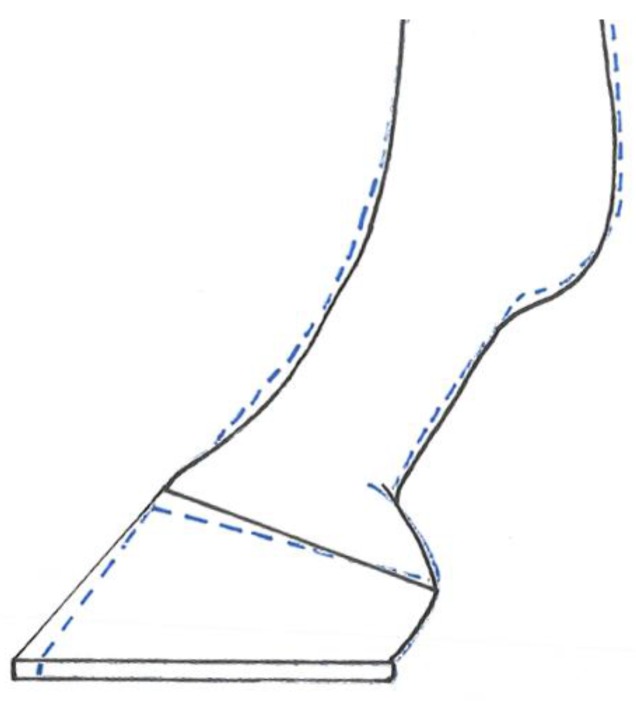
Pre-trimming (solid black line) and post trimming (blue dotted line); the solid line also represents the increased vertical orientation observed post-shoeing/trimming. Note the more upright angle of both the heel angle and DHW angle, and the shortening of the toe.

Changes reported in the lateral view suggest that angular modifications of the distal limb are occurring at the level of both the MCP and DIP joints. The reduced DHWL, increased DHW and heel angle, the decrease in the weight bearing length, and increase in the palmar coronary band height, suggest that although the toe shortened, the length of the heel was not altered during the shoeing/trimming procedure. Following trimming, the weight bearing length decreased in the anterior view, whilst the medial and lateral wall lengths, as measured from the digital images, increased. The changes which occurred in the MCP and DIP joint angulations across the lateral view could also explain the increase in medial and lateral wall lengths from the anterior view images. We believe that the decrease in the coronary band lengths observed from both the lateral and anterior view 2D images occurred as a result of the change in positioning of the hoof capsule post-shoeing/trimming. The dorsal wall shortens by a relatively greater amount than the heels, which results in the hoof assuming a more vertical orientation. This is supported by the differences found between the lateral and medial hoof wall length; 73% of participants had a longer lateral wall pre-shoeing, and this increased to 88% of the population post-shoeing. Furthermore, the mean difference between the two sides increased following routine shoeing, from 7% to 10%. Guidance in achieving mediolateral balance of the equine foot refers to the trimming of the medial and lateral walls, to ensure that the live sole of the foot is level with the ground [35] and the hoof is in balance with the limb column [27], as opposed to the postulation that balance is attained through the attainment of symmetrical wall lengths [29]. More recent studies have found that subtle asymmetries manifested as a more upright medial wall and a more angled lateral wall are common within the domestic horse population [8,36,37,38,39] and reflect the lateral landing and unrollment pattern of the foot observed in sound horses [17,40]. Our results support the practice of trimming according to the live sole, with a more inclined conformation, without altering the mediolateral balance of the foot, to promote soundness and not change the wall lengths. Use of the live sole as guidance ensures that following lateral landing and unrollment, loading of the foot produces equal pressure across the circumference of the foot to the ground, through the anatomical structures in the distal limb [10]. The differences reported in the medial and lateral wall lengths can also reflect the difference in the angles between the walls and the weight bearing surface of the foot (Figure 3).

Pre-shoeing, only 31% of horses presented with a greater LHA, though this decreased to 12% post-shoeing. The more slanted lateral wall presented a more acute angle with the sole compared to the angle on the more upright medial wall, which was more obtuse, by between 1° and 7°. Approximately 25% of participants presented with a longer medial than lateral wall pre-shoeing, suggesting that the loading pattern that they exhibited differed from normal lateral landing and unrollment. These differences potentially infer a more medial landing posture, indicating that some degree of low level lameness was inherent in the study population; a potential inherent career risk of riding school horses [41]. Further to the influence of the loading pattern, mediolateral balance can also be influenced by the individual farrier, with significant differences previously observed between individuals [42]. The current study did not evaluate the influence of individual farriers and therefore this could account for some of the differences found.

Interestingly, although MHAs and LHAs changed post-shoeing, these changes were not significant. A similar number of horses presented with an increase and decrease in LHA; however, a greater proportion of the sample (58%) demonstrated an increase in MHA post-shoeing. The lack of significant results is attributed to the large individual variations seen between the horses examined.

The hoof pastern axis (HPA) is defined by plotting a line down from the middle of the metacarpophalangeal joint, through the centre of the proximal and distal interphalangeal joints, and through the axis of the phalanges [10,26]. A straight alignment is accepted as ideal and considered optimal for physiological function [11,42]. The shortening of DHWL through trimming of the toe within the current population, accompanied by an increase in both the heel angle and the coronet band at the heels, placed the hoof capsule in a more vertically orientated position. At the same time, there is a consequential increase in the heel angle and a corresponding reduction in HPA displacement, combined with a concurrent increase in the fetlock joint angle and decrease (not significant) in vertical limb displacement. These changes in joint angulations suggest that a four to six week shoeing interval contributes toward promoting consistency in HPA through addressing the fetlock joint angle [6] and vertical displacement. However, for these horses, the four to six week interval did not stop the foot from becoming broken backwards (where DHWA is more acute than the angle of the dorsal pastern.), and so the changes observed may just be mechanical and a static response to trimming of the toe. If the foot moves towards a more broken back angulation throughout the duration of even a four to six week shoeing interval, where a more traditional six to eight week interval is used, the broken back angulation might reach a level whereby loading of the navicular region and the suspensory apparatus, specifically the DDFT, may be detrimental and enhance the risk of injury.

The forelimb CoP is known to deviate in a palmar direction across an eight week shoeing interval, whilst breakover remains relatively consistent [4,17]. The centre of pressure (CoP) within the current study was predicted to be, on average, 0.5 cm in a palmar direction to the point of rotation of the toe across the cohort, which is less than half of that of the 1.3 cm observed over an eight-week interval by Van Heel et al. [4]. The shoe of a shod horse prevents the wear of the toe, but not of the heel, and therefore, as the toe lengthens, the angle of both the toe and the heel decrease, resulting in the palmar movement of the CoP, and an increased loading in the DIP joint and the DDFT.

Moleman et al. [6] identified similar growth patterns at the toe across an eight week shoeing interval, but found none at the heel and no significant change in PIP joint moment. Consequentially, the change of motion is thought to be located in the DIPJ, increasing loading on the DDFT and navicular bone, which act to stabilise the DIPJ in order to maintain a dynamic equilibrium during locomotion [6,11,43]. The shortening of the DHWL achieved during trimming reverses these mechanics through the correction of deviation of the CoP away from the central foot axis [44], to a more dorsal location, and thereby reduces the load on the suspensory apparatus. The reduced palmar shift in the CoP observed within the shorter four to six week interval examined here, suggests that this time frame would exert a protective effect on DIPJ and DDFT loading, compared to longer shoeing intervals.

### Limitations

The current study applied a pragmatic and observational research approach to investigate the impact of shoeing practices on hoof morphology in riding school horses. Whilst the study has strong external validity, it should be noted that the real-world design possesses a number of limitations, and therefore, the results should be interpreted with caution. Many factors can influence hoof growth. These include, but are not limited to, horse breed, height, diet, disease, time of year, the environmental effects of pasture quality, and lameness. The horses integrated here were subject to consistent management practices and were considered fit for riding school work by experienced equestrian professionals and the supporting veterinary team. The presence of low grade lameness has been previously identified in riding school horses to affect loading patterns and, subsequently, hoof growth, which could explain some of the variation observed within our results [41]. Subjects did vary in breed and height, which could have influenced the results obtained. Similarly, although every effort was made to ensure that horses were standing square prior to digital photography through the use of handlers and the positioning grid [2] in order to prevent positional rotation of the distal limb and foot, there is a possibility that the measurements taken integrate some degree of rotation. Force plate analysis is recommended for future studies, to accurately assess that horses are standing square and that equal loading is exhibited in each of the four limbs. Measurements of both the right and left hooves are also advocated in future research, to facilitate comparative analysis. Previous research [42] has associated morphometric differences in hoof measurements post trimming with individual farrier techniques and personal interpretations of the HPA. A team of farriers was utilised here. Furthermore, all farriers worked under the direction of one lead farrier and trimmed and shod horses according to their instructions, promoting a more consistent approach, which should limit the impact of individual technique. However, to ensure that the changes observed here are not related to individual farriery techniques, we would advise future research to use a consistent farrier or to assess the impact of individual farriers within data analysis.

## 5. Conclusions

After shoeing, hoof-surface interface interactions result in dynamic responses in the hoof promoting adaptation over time within the hoof’s structural components, affecting linear and angular measurements. In the shod horse, the presence of the shoe prevents toe wear. Therefore, the adaptation which occurs over time will be influenced by the shoe. Even with shoeing intervals of four to six weeks, changes are observed in key parameters associated with foot balance. Our results suggest that, for the majority of horses, the weight bearing length of the foot increases (as the toe grows), causing a decrease in the heel angle and an increase in hoof angle displacement as the hoof pastern axis becomes more broken backwards, negatively influencing dorsopalmar balance. Changes also occur in the dorsal wall associated with loading during locomotion; in the shod horse, foot placement demonstrates a lateral-medial unrollment pattern. Therefore, increased loading may occur in the lateral hoof wall, which would result in lateral hoof wall length increasing and the lateral angle decreasing over time. In contrast, and in agreement with previous research [6], a four to six week interval retains consistency within fetlock joint angles (−0.10° ± 10.5°) and vertical displacement (0.65 ± 2.6) of the HPA, positively influencing dorsopalmar balance, which should aid in the prevention of tendinopathies. Therefore, overall, we have to reject our original hypothesis that no changes would occur across HPA and hoof morphometric measurements during a four to six week shoeing interval. Further work is required to confirm these findings across more horses and with a comparison between different shoeing intervals, accompanied by an additional lameness and distal limb conformation evaluation. A comparison would also be worthwhile to evaluate whether similar changes are observed pre- and post-trimming within the unshod horse and with differing workloads and farriers. Horses within the current study were predominantly working on a soft surface, resulting in reduced loading forces on the hoof [1], and as such, the results from our cohort might underestimate the effect seen in horses working on more variable or harder terrain.

Caution is advised in the interpretation of these results, as a high degree of inter-subject variation was found across the measurements undertaken and as a number of variables could not be controlled due to the horses’ working schedule. This variation could represent individual conformation differences in the hoof or distal limb, could be a cumulative result of previous farrier treatments [42], or may be a result of confounding variables. Based on our findings, we would recommend that horses are considered as individuals when determining their ‘optimal’ shoeing interval, but that the length of this period should not exceed six weeks. In addition, whilst we acknowledge that much published work still recommends that the ‘perfect’ foot should be symmetrical, it appears that this is not commonly observed within general riding horses, especially when they are shod. Therefore, we would respectfully suggest that the traditional aim of farriery to produce a balanced foot is adapted to a horse’s specific functional requirements, in order to optimise performance and longevity. This can be achieved by shifting the focus to create a balanced foot with due consideration of the conformation of the individual’s distal limb and the parallel alignment of the solar and weight bearing surfaces.

## Figures and Tables

**Table 1:** Location of anatomical markers.

1. Midpoint of dorsal hoof wall, proximal margin	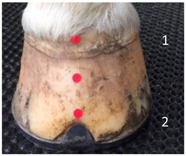
2. Midpoint of dorsal hoof wall, distal margin	
3. Dorsal aspect of the radiocarpal joint
	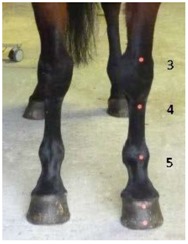
4. Dorsal aspect of the carpometacarpal joint	
5. Dorsal aspect of the metacarpal phalangeal joint	
6. Lateral aspect of the radiocarpal joint	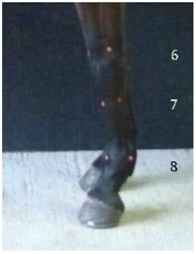
7. Lateral aspect of the carpometacarpal joint	
8. Lateral aspect of the metacarpal phalangeal joint	

**Table 2:** Angular and linear morphometric variables measured from the 2D lateral view images of the hoof [22,23].

Lateral Hoof Measurements		
Variable	Abbreviation	Description
Dorsal hoof wall length	DHWL	Length of dorsal hoof wall from hair line at the coronary band to ground level
Weight bearing length lateral	WBL-L	Length from the dorsal to the palmar point of the hoof wall in contact with the ground surface
Coronary band length	CBL	Length from the dorsal to the palmar point of the coronary band
Dorsal hoof wall angle	DHWA	Angle between the dorsal hoof wall and the ground plane
Heel angle	HLA	Angle between the palmer aspect of the hoof wall and the ground surface
Dorsal coronary band height	DCBH	Vertical height between the dorsal region of the coronary band and the solar plane
Palmer coronary band height	PCBH	Vertical height between the palmer region of the coronary band and the solar plane

**Table 3:** Angular and linear morphometric variables measured from the 2D dorsal view images of the hoof.

Dorsal Hoof Measurements		
Variable	Abbreviation	Description
Weight bearing length dorsal	WBL-D	Coronary band width between the lateral and medial hoof walls at the distal region of the hoof
Coronary band width	CBW	Support length between the lateral and medial hoof walls at the proximal region of the hoof
Medial dorsal hoof wall length	MDHWL	Length of the medial hoof wall from hairline to ground
Midline dorsal hoof wall length	CDHWL	Length of the hoof wall at the midpoint of the hoof from hairline to ground
Lateral dorsal hoof wall length	LDHWL	Length of the lateral hoof wall from hairline to ground
Medial hoof angle	MHA	Angle between the medial hoof wall and solar plane
Lateral hoof angle	LHA	Angle between the lateral hoof wall and solar plane

**Table 4:** Angular and linear morphometric variables measured from the 2D lateral view of the limb.

Lateral Limb Measurements		
Variable	Abbreviation	Description
1. Vertical displacement (Yellow)	VD	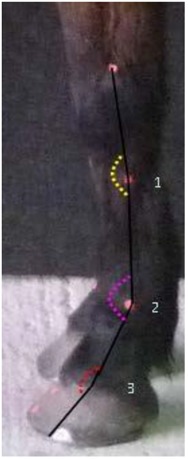
2. Fetlock joint angle (Pink)	FJA	
3. Hoof angle displacement (Red)	HAD	

**Table 5:** Differences between hoof measurements pre- and post-farriery. Bold probability values denote statistical significance. (Length, width and height in cm; angles in degrees).

	Variable	Mean ± Standard Deviation		*p* Value	Increase/Decrease
		Pre-Farriery	Post-Farriery		
***Lateral View***	DHWA	52.1° ± 3.47°	54.36° ± 3.99°	***p* = 0.0001**	Increased
	HLA	45.49° ± 7.59°	49.96° ± 5.55°	***p* = 0.0001**	Increased
	DHWL	7.81 ± 1.35 cm	7.56 ± 0.91 cm	*p* > 0.05	Decreased
	WBL-L	11.58 ± 1.16 cm	11.04 ± 1.4 cm	***p* = 0.0001**	Decreased
	CBL	10.88 ± 0.96 cm	10.15 ± 1.09 cm	***p* = 0.0001**	Decreased
	DCBH	7.22 ± 1.21 cm	7.43 ± 0.78 cm	*p* > 0.05	Increased
	PCBH	2.70 ± 0.63 cm	3.24 ± 0.56 cm	***p* = 0.0001**	Increased
***Anterior View***	CBW	5.39 ± 1.00 cm	5.09 ± 1.09 cm	***p* = 0.05**	Decreased
	WBL-D	6.84 ± 1.44 cm	6.14 ± 1.29 cm	***p* = 0.001**	Decreased
	CDHWL	3.87 ± 0.61 cm	4.14 ± 0.94 cm	*p* > 0.05	Increased
	MDHWL	3.57 ± 0.69 cm	3.84 ± 0.74 cm	***p* = 0.03**	Increased
	LDHWL	3.72 ± 0.68 cm	4.09 ± 0.80 cm	***p* = 0.009**	Increased
	MHA	78.96° ± 5.81°	80.17° ± 5.41°	*p* > 0.05	Increased
	LHA	73.17° ± 4.20°	72.79° ± 4.07°	*p* > 0.05	Decreased
***Lateral limb***	HAD	189.49° ± 4.89°	183.28° ± 2.89°	***p* = 0.0001**	Decreased
	FJA	212.71° ± 8.03°	212.81° ± 8.48°	*p* > 0.05	Increased
	VD	184.04° ± 2.72°	183.39° ± 2.11°	*p* > 0.05	Decreased

**Table 6:** Significantly correlated variables pre- and post-farriery. Bold regression co-efficient values denote strong (>0.75) relationships.

Variables		Pre-Farriery		Post-Farriery	
		*r co-eff*	*p* Value	*r co-eff*	*p* Value
DHWA	WBL-L	−0.39	0.050	−0.45	0.022
LHA	PCBH	0.42	0.031	-	-
DHWL	CBL	0.57	0.002	-	-
DHWL	DCBH	**0.93**	0.0001	0.40	0.043
DHWL	PCBH	**0.75**	0.0001	0.65	0.0001
DHWL	LDHWL	-	-	0.50	0.009
WBL-L	CBL	0.68	0.0001	**0.86**	0.000
WBL-L	PCBH	0.46	0.019	-	-
WBL-L	CBW	0.66	0.0001	0.46	0.018
WBL-L	WBL-D	0.67	0.0001	-	-
WBL-L	LDHWL	-	-	0.38	0.053
CBL	DCBH	0.59	0.001	-	-
CBL	PCBH	0.61	0.001	-	-
CBL	CBW	0.59	0.002	0.52	0.007
CBL	WBL-D	0.65	0.0001	-	-
CBL	CDHWL	-	-	0.41	0.040
CBL	MDHWL	-	-	0.41	0.036
CBL	LDHWL	-	-	0.50	0.010
DCBH	PCBH	**0.84**	0.0001	-	-
PCBH	HAD	−0.41	0.039	-	-
CBW	WBL-D	**0.95**	0.0001	**0.87**	0.0001
CBW	CDHWL	0.54	0.005	**0.78**	0.0001
CBW	MDHWL	0.67	0.0001	**0.76**	0.0001
CBW	LDHWL	0.58	0.002	**0.82**	0.0001
WBL-D	CDHWL	0.53	0.005	**0.79**	0.0001
WBL-D	MDHWL	0.64	0.0001	**0.76**	0.0001
WBL-D	LDHWL	0.53	0.006	**0.79**	0.0001
CDHWL	MDHWL	**0.91**	0.0001	**0.93**	0.0001
CDHWL	LDHWL	**0.86**	0.0001	**0.92**	0.0001
CDHWL	LHA	0.44	0.026	-	-
MDHWL	LDHWL	**0.91**	0.0001	**0.92**	0.0001
MDHWL	MHA	−0.41	0.040	-	-
FJA	VD	−0.70	0.0001	-	-
MHA	FJA	-	-	−0.44	0.025
VD	MHA	-	-	0.51	0.008
HLA	VD	-	-	0.41	0.035
